# Comparison of Different Materials for Self-Pressurized Vitrification of Feline Oocytes—First Results

**DOI:** 10.3390/ani11051314

**Published:** 2021-05-03

**Authors:** Lorena Fernandez-Gonzalez, Jan Huebinger, Katarina Jewgenow

**Affiliations:** 1 Department of Reproduction Biology, Leibniz Institute for Zoo and Wildlife Research, Alfred-Kowalke-Str. 17, D-10315 Berlin, Germany; jewgenow@izw-berlin.de; 2 Department of Systemic Cell Biology, Max-Planck-Institute of Molecular Physiology, Otto-Hahn-Str. 11, D-44227 Dortmund, Germany; jan.huebinger@mpi-dortmund.mpg.de

**Keywords:** high pressure freezing, self-pressurized rapid freezing, cat oocytes, cryobanking, felids, ICSI, titanium, aluminium, silver

## Abstract

**Simple Summary:**

In endangered species, the remaining individuals live in small isolated groups. Cryobanking of gametes represents the possibility of saving genetic variability, and increasing diversity by being the bridge between populations. Therefore, it is an urgent need to provide reliable methods in order to preserve these valuable resources under well-established conditions. Self-pressurized freezing is a successful technique that can be performed in remote areas without special equipment, which is a requirement to rescue material from wild specimens. In this study, we aim to check the efficacy of this method to vitrify felid oocytes and to find a suitable material for this procedure. One hundred and eighty nine oocytes from domestic cat were frozen in three different material tubes: Aluminium, silver, and titanium. Aluminium presented toxic effects that were corroborated by the moderately lower cleavage rates (14.3%), followed by silver (18.2%). Furthermore, in the literature, silver has been related to biological alterations in the cells. A minor tendency for higher rates, in both maturation after warming of the oocytes (55.6%) and cleavage after fertilisation (20%), were obtained with titanium, nevertheless there was no statistical difference between the outcome. More studies are needed to improve the method and increase embryo development after warming.

**Abstract:**

Cryobanking is a crucial part on species conservation. Nowadays, there is no suitable protocol for vitrification of feline oocytes. Self-pressurized rapid freezing of different cell types proved to mimic the advantages of high pressure freezing. As this method could also be applied for gamete rescue under field conditions, the aim here was to analyse the impact of self-pressurized vitrification on feline cumulus-oocyte-complexes (COCs) and to determine the appropriate material. Therefore, COCs of domestic cat were randomly vitrified (n = 189) in metal tubes of different materials: Aluminium, silver, and titanium. No significant differences were found on oocytes’ competence after thawing. On average, 44% of the COCs presented normal morphology and 48.2% of them showed a polar body after in vitro maturation (IVM) and were subsequently fertilised. Aluminium tubes were positive on toxicity tests, producing the lowest cleavage rates. Silver tubes showed no toxic effect, but the cleavage rate was lower than with titanium tubes, and a previous association with embryotoxicity and biological alterations makes us aware of its indiscriminate use. Titanium seems to be the only inert material of them, presenting a slightly higher maturation (55.6%) and cleavage (20%) rates. Nevertheless, more studies should follow to increase embryo competence after warming.

## 1. Introduction

Cryopreservation of gametes is an important part of assisted reproduction techniques (ARTs), which are recommended to be applied for species conservation. Biobanking of gametes is employed when the species are highly threatened and the remaining groups do not have enough individuals to ensure healthy genetic diversity [1]. Most of the felids are currently classified as threatened or near-threatened by the IUCN Red List [2]. Therefore, it is a matter of necessity to store germinal cells under well-established conditions [3] and for this concern the domestic cat, *Felis catus*, serves as a model species.

Vitrification of oocytes is a successful and well-established procedure for species such as humans [4] or cattle [5], but novel approaches to improve the methodology are still the focus of many research groups. For example, in mouse the use of a laser pulse for ultra-rapid warming increased oocyte survival until nearly 100% [6]. In particular, vitrification in cats is still considered an experimental technique and traditional protocols bring insufficient developmental rates, showing that only oocytes from 14.1 to 38.7% are able to undergo successful IVM after vitrification and warming [7,8,9,10,11,12].

Self-pressurized rapid freezing is a new and low-cost cryofixation method that instantly arrests all the biochemical and physiological processes in the cells [13]. This method allows getting the advantages of high pressure freezing by using self-closed metal tubes that are directly plunged into the liquid nitrogen, avoiding the use of special equipment [14]. High pressure freezing was in principle created for electron microscopy preparations [15,16] since it allows the maintenance of the original microstructure at cooling and warming rates that might not be achievable under atmospheric conditions. The also called “rapid freezing” has been used in several fields, even in the food industry [17].

Until now, the cryotop is the most used device for vitrification of oocytes [18], but the use of closed metal tubes might present many advantages [19]. First of all, due to a higher thermal diffusivity of the metal when compared to the plastic [20], freezing in metal tubes provides higher cooling and warming rates than freezing in plastic devices. Furthermore, due to the smaller size of the metal tubes, they need much less storage space per stored volume. Moreover, having a totally closed system guarantees no contamination transmission through the liquid nitrogen [21]. Lastly, it is hypothesised that the use of high pressure freezing could be equivalent to a decrease of 20% of the cryoprotectant (CPAs) solutions [22], minimising toxic effects on the samples.

Our aim here was to find out whether the combination of vitrification and self-pressurized rapid freezing of cat oocytes could improve oocyte survival compared to the standard vitrification protocols. Three materials were chosen based on intrinsic properties: Aluminium, recently introduced in electron microscopy as the softest material, is easy to close with pliers; silver, also used for microscope analysis, is still soft but more resistant towards clipping; whereas titanium, regarded as inert material is not only very expensive, but the hardness aggravates the closing procedure. Due to previous indications of toxic effects of aluminium and silver, we also tested the impact of the three materials on cell survival even though the contact during vitrification and warming processes is restricted to very short periods of time.

## 2. Materials and Methods

All chemicals are purchased from Sigma-Aldrich (Taufkirchen, Germany) if not stated otherwise.

### 2.1. Oocyte and Sperm Collection

Gonads were obtained from Berlin Animal Shelter after routine gonadectomy. Surgeries were performed independently of the development of this study. Ovaries were transported in Minimum Essential Medium Eagle HEPES modification (HEPES-MEM) supplemented with 1:100 (v:v) Antibiotic Antimycotic Solution and 3 mg/mL BSA, while testes were transported without medium, both stored in an expanded polystyrene box. Samples were used either upon arrival on the lab or after keeping them refrigerated at 4 °C overnight. Ovary surfaces were sliced with a scalpel blade in washing medium (WM) composed of Medium 199 (M199) with Earle’s salts supplemented with 0.1 mg/mL cysteine, 3 mg/mL BSA, 0.055 mg/mL gentamicin, 0.15 mg/mL L-glutamine, 0.25 mg/mL sodium pyruvate, 0.6 mg/mL sodium lactate, and 1.4 mg/mL HEPES [23] in 6 cm petri dishes (Sarstedt, Nürnbrecht, Germany). High quality COCs, referred to those with a dark and homogenous ooplasm and several granulosa cell layers surrounding it, were collected under an inverted stereomicroscope (Nikon SMZ 745T, Nikon, Düsseldorf, Germany).

Vas deferens and cauda epididymis were dissected from cat testes and carefully minced in prewarmed M199 to allow the release of the spermatozoa. The quality of the sample was quickly estimated at 20× magnification on a phase contrast microscope (Axioskop, Carl Zeiss, Jena Germany). The solution was centrifuged at 300× *g* during 8 min and after discarding the supernatant, fresh prewarmed M199 was added to the pellet.

### 2.2. Toxicity Tests

For toxicity assessment, firstly sperm cells were used due to larger availability. Basically, the test consisted of the coincubation of these cells with the CPAs present in the freezing media, 10% Me2SO and 10% ethylene glycol (EG) in Dulbeco’s PBS (DPBS) diluted 1:1 with the sperm solution, thus 10% of CPAs concentration in total. Two groups were measured: Sperm cells mixed with either fresh CPAs (control) or with CPAs that have been previously preincubated in the tubes (treatment). Motility was assessed at 0 and 4 min with the computer-assisted software AndroVision^®^ (Minitube, Germany) by analysing at least 1000 sperm cells in each test. The toxicity test was repeated with COCs in the cases where a toxic effect was suspected. In this case, the vitrification solution was used: 20% Me2SO, 20% EG, 20% FCS, 10% Ficoll PM-70, and 1.5M trehalose dissolved in DPBS, corresponding to 40% of CPAs concentration. Thus, COCs were inserted in the tubes with WM (control) or together with the vitrification solution but for a shorter time, 2 min. There was no control on the effect of CPAs alone with COCs since this possibility has been previously studied, demonstrating no toxic issues [24]. Later oocyte competence was analysed in terms of maturation success and cleavage after fertilisation.

### 2.3. Vitrification and Warming

Directly after collection, batches of 2–3 immature COCs were vitrified with our 3-step protocol [25] slightly modified. Briefly, oocytes are settled for 3 min on the equilibration solution prepared with 7.5% Me2SO and 7.5% EG diluted in DPBS, followed by a second equilibration with higher concentration of CPAs, 10% Me2SO, and 10% EG in DPBS. Then, within 30 s COCs are shortly incubated in a vitrification medium, with 20% Me2SO, 20% EG, 20% FCS, 10% Ficoll PM-70, and 1.5M trehalose dissolved in DPBS, inserted in the metal tubes with a micropipette (The Stripper^®^, BioTipp, Waterford, Ireland) where the stripper tip was adapted to the tube diameter size, and both sides are firmly closed with pliers. Tightly closed tubes were directly plunged into liquid nitrogen.

Three different tube materials were tested: Aluminium with inner/outer diameter of 0.6/0.3 mm, silver with 0.6/0.3, and titanium with 0.51/0.35 (GoodFellow GmbH, Hamburg, Germany), cut to a length of 10 to 15 mm.

For thawing, tubes were rapidly taken out of the liquid nitrogen and immersed in a 3.5 cm petri dish with 5 mL of thawing solution pre-warmed at 38.5 °C, consisting of 30% Ficoll PM-70 and 1 M trehalose diluted in DPBS. Both ends were opened either with scissors or forceps, depending on the material, and the COCs were flushed out into the thawing solution and kept there for 30 s. Next, they were moved for equilibration to a drop of thawing solution diluted 1:1 in DPBS for 5 min, followed by the exposition in groups to 400 µL of recovery medium, WM supplemented with 10% Ficoll PM-70 covered with mineral oil (Reproline Medical, Waterford, Ireland) for 2 h.

### 2.4. Calculation of Thermal Diffusivity Limited Cooling Rates

Heat-flux limited cooling from a surface can be calculated based on the equation for the rate of heat flow

(1) $$\frac{dQ}{dt}=-A\frac{T}{L}$$

with the surface area (*A*), thermal conductivity (κ) (water: app. 0.6 Wm^−1^K^−1^), the initial temperature difference (Δ*T*: app. 233 °C between a sample at 37 °C and liquid nitrogen at −196 °C). The thickness (*L*) was the inner radius of the tubes or of a half-sphere for a drop of solution on a cryotop.

The heat flow *Q* can also be described as:

(2) Q=c×m×T

with c being the heat capacity (water: 4.2 Jcm^−3^K^−1^) and m the mass of the sample.

Combined, this results in

(3) $$\frac{dT}{dt}=-A\frac{T}{cmL}$$

which gives the maximal possible conductive cooling rate in the center of the sample.

### 2.5. IVM and Fertilisation

After the recovery time, COCs were washed once in WM and settled in 400 µl of IVM medium prepared with WM supplemented with 0.02 IU hLH/mL (L6420) and 0.05 IU hFSH/mL (Ferring, Kiel, Germany) and covered with mineral oil for 24 h at 38.5 °C and 5% CO_2_ under humidified atmosphere. COCs were then released of the granulosa layers by gently pipetting with a micropipette.

Fertilisation was performed through intracytoplasmic sperm injection (ICSI). In brief, a 6 cm petri dish was prepared with drops of 5 µL of WM supplemented with 0.5 mg/mL HEPES to hold the oocytes and two 10 µL drops of polyvinylpyrrolidone (PVP) diluted 1:2 in WM were disposed for the sperm solution, everything covered with mineral oil. Under 200× magnification on an inverted microscope (Axiovert 100, Carl Zeiss, Jena, Germany), oocytes were examined for the extrusion of the polar body as a sign of metaphase II. When it was present, an immobilised sperm cell with normal morphology was injected in the ooplasm. Immediately, presumptive zygotes were settled in 400 µL of embryo culture medium, consisting of Ham’s F-10 supplemented with 0.075 mg/mL L-glutamine, 0.1 mg/mL streptomycin, 5% FCS, and 100 IU/mL penicillin, and covered with mineral oil. They were cultured at 38.5 °C and 5% CO_2_ under reduced oxygen (5%).

### 2.6. Fluorescence Staining

The oocytes without the polar body were dried on a glass slide and fixed with 96% of ethanol for at least 24 h. The nucleus stage was evaluated by staining with propidium iodide (10 µg/mL) diluted in DPBS and the assessment proceeded under a 40× magnification on an inverted fluorescence microscope (Axiovert 200, Carl Zeiss, Jena, Germany).

### 2.7. Statistics

Statistical analysis was performed with the software InStat^®^ (InStat, GraphPad Software, La Jolla, CA, USA). decrease in sperm motility in the toxicity test, as likeas the numbers of in vitro matured oocytes, cleaved oocytes, and embryos both in toxicity tests and in vitrification procedures were analysed with two-tailed contingency tables with Fisher’s Exact Test. Significance was considered when the *p*-value was lower than 0.05.

## 3. Results

### 3.1. Toxicity Test

The assay performed with sperm cells, owing to a higher accessibility, showed that in the control groups, the motility of sperm cells after 4 min incubated in DPBS with CPAs was between 60–80% compared to the motility at 0 min. In the treatments, when DPBS with CPAs was preincubated with aluminium tubes a further drop to about half until approximately 38% of the original motility was determined. The silver and titanium tubes did not show such a pronounced effect. Incubation of sperm cells in DPBS with CPAs preincubated with both types of tubes resulted in no significant further loss of motility compared to the control groups itself, thus this decrease was only around 8% higher.

Based on the observed effect of aluminium tubes in combination with CPAs, the toxicity test in these tubes was performed with COCs, as well. Twenty-two COCs were used as the control and 23 as the treatment, for further development competence analysis. It was observed that a significantly higher number of oocytes was mature in the first group (*p* = 0.03, Fisher’s exact test) represented by 13 oocytes (59.1%) against only three in the second (13%). Fertilisation by ICSI produced three embryos in the control group (23.1%) developing until the morula stage, while no embryo cleavage was observed in the group of the oocytes incubated with CPAs in the tubes.

### 3.2. Oocyte Vitrification, IVM, ICSI, and Fluorescence Staining

In total, 189 oocytes were vitrified, distributed as follows: In the group of oocytes frozen in aluminium tubes n = 61, in the silver group n = 63, and in the titanium group n = 65 (Table 1). After warming and IVM, a morphology analysis was performed. Oocytes were classified into either normal appearance or those which showed any damage. Around half of the oocytes presented a normal morphology with no statistical difference between the groups. Moreover, no statistical difference was observed after IVM. If all the oocytes are taken together and maturation is compared to our previous vitrification results with cryotop as a freezing device [10], no difference was found between the two approaches.

**Figure 1:**
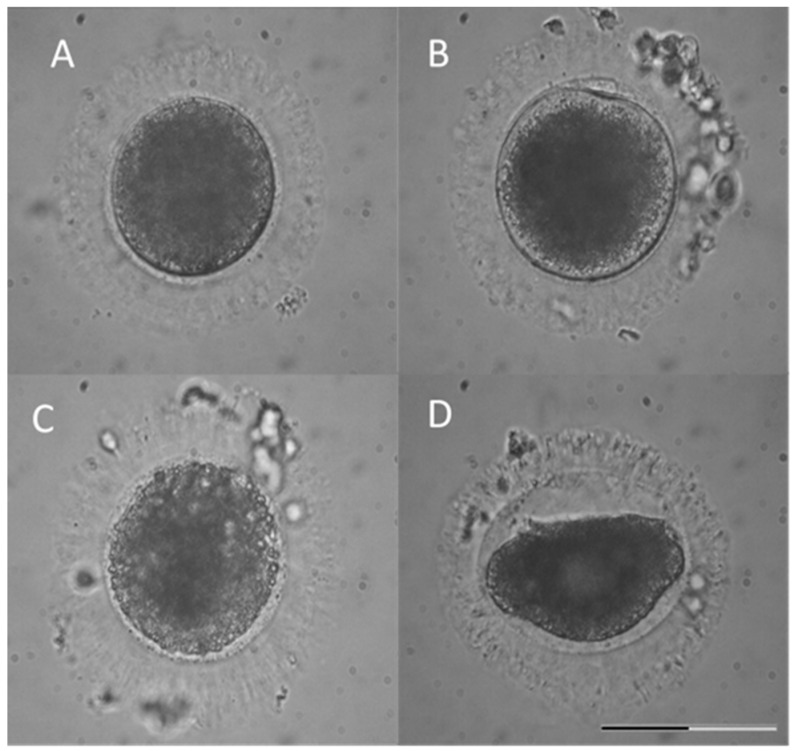
Morphological appearance of the oocytes after warming and IVM. Classified as: (**A**) Normal oocyte, (**B**) normal and mature oocyte, (**C**) oocyte with damaged membrane, and (**D**) shrunken oocyte. Scale bar = 100 µm.

In Figure 1, morphological aspects are presented. Some of the damaged oocytes normally expanded after a short recovery time but were still characterised by visible membrane defects (Figure 1C). Another group was severely damaged, since they did not even expand to the original size after warming (Figure 1D). All these oocytes together with the ones that did not extrude a polar body (Figure 1A) were subjected to nuclear stage analyses via fluorescence.

**Figure 2:**
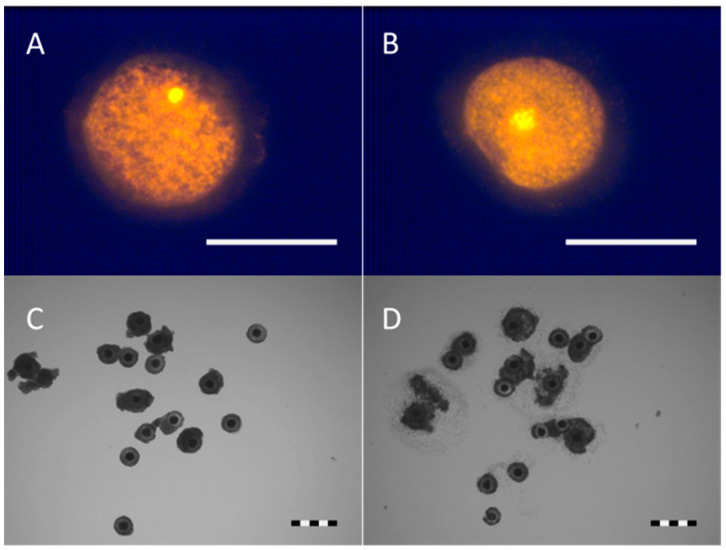
Oocytes stained with propidium iodide are shown on a fluorescence microscope (**A**), a GV, and (**B**) an early GVBD, scale bar = 100 µM. Oocytes frozen in titanium tubes (**C**) before and (**D**) after IVM, scale bar = 500 µM.

Staining of fixed oocytes with propidium iodide for the nucleus stage allowed for separation into different categories (Table 2): Oocytes in germinal vesicle stage (GV), and those that started the resumption of meiosis (progress on meiosis). In all three groups, about 60% of the immature oocytes remained at the GV stage (Figure 2A), and again no differences between the tubes were observed. Oocytes which were progressing in meiosis were either characterised by a germinal vesicle break down (GVBD, Figure 2B) or metaphase I (MI). A small percentage of oocytes were degenerated and/or the maturation stage could not be assessed.

The developmental competence of intact oocytes after warming is represented in Table 3. The highest percentage of maturation was found in the titanium group with 15 oocytes (55.6%) presenting a polar body after warming and IVM, followed by about 45% in the aluminium and silver group, although the differences for maturation and cleavage rates were not significant. Embryo development achieved the 6-cell stage in the aluminium group, while in silver and titanium was blocked at the 4-cell stage.

## 4. Discussion

The use of new approaches to improve survival rates seems to be necessary in felids, since so far standard vitrification protocols offer repeatedly poor results. It is known that one of the biggest disadvantages of vitrification is the high concentration of CPAs that might cause a toxic effect on the cells. Theoretically in this case, less pronounced ice formation should happen inside of the closed tube due to the expansion of the water prior to freezing and cryoprotective agents could be lowered. It is estimated that the application of high pressure supports a reduction of 20% of CPAs [22]. In our preliminary study, this theory was not confirmed. The decrease of CPAs concentration to 20% resulted in extreme damage of the COCs and produced 0% of the maturation rate (results not presented). It remains open whether a lower reduction of CPAs could be enough to guarantee cell survival. We hypothesised that as previously shown, some hexagonal ice starts to grow inside of the tubes [13], and a high concentration of CPAs is needed to prevent the ice crystal formation inside of the cells [26].

The cylindrical aqueous sample can be theoretically cooled with 1400 °C/s (diameter: 0.3 mm) or 1000 °C/s (0.35 mm) in the center. Despite the larger volume used in self-pressurized rapid freezing, this is very comparable to a 0.1–0.5 µL drop of liquid forming a half-sphere on a cryotop (400–1900 °C/s, see Section 2.4. for calculations). The thermal conductivities of the metal surrounding are 2–4 orders of magnitude higher and are thus not limiting for the cooling rates of the sample. However, these rates are theoretical, since they assume a sufficiently high cooling rate at the surface. If a sample is plunged into liquid nitrogen a layer of gaseous nitrogen forms at its surface (Leidenfrost effect), which has a low thermal conductivity (app. 0.04 Wm^−1^K^−1^). Real cooling rates are thus lower and unknown. To our knowledge, a temperature measurement in such small samples with the sufficient time resolution and without changing the sample, e.g., by a conducting thermocouple, has not been done.

In vitrification procedures, cells are in contact with toxic solutions and go through osmotic changes where the experience and speed of handling is related with the success of the procedure. Here, working with opaque tubes makes the loading of the cells more challenging. In a very short time, cells have to be inserted in the middle of the tube so they are not crushed when the sides are closed with pliers. For warming, in contrast with open devices both extremes of the tubes need to be opened, in soft materials it is possible to do this step with forceps but in hard materials the operator needs to cut the sealed part. It might be necessary to increase the length of the tubes due to the risk of a secondary crushing of the cells.

A successful IVM was observed in about 20% of the oocytes vitrified in tubes. These rates are only slightly lower than those obtained with the same media and the cryotop device in previous studies, where the maturation rate was up to 24.5% [10]. The fact that produced cryodamages are similar in metal tubes and cryotops was previously stated with other cell lines [19]. Taking into account the differences between the methods (materials, intrinsic characteristics, volume, etc.) this could mean that ice formation is not the crucial factor for the success of this cryopreservation, as it was previously showed that the use of slush nitrogen for freezing is also not improving the survival in cat oocytes [10]. Nevertheless, the biggest difference is found on embryo cleavage and development. Here, a slightly higher result was found with titanium tubes obtaining 20% of cleavage with embryos developing until the 4-cell stage, but in the previous study we presented 28.6% of cleavage with the cryotop with 30% of embryos achieving the morula stage [10]. While IVM rates after warming are in accordance with previous publications in felids [7,11], embryo competence seems to be triggered as in other novel approaches [27].

Furthermore, in this study, the morphology assessment showed that only around half of the oocytes might survive the freezing-warming procedure. We can consider the oocytes with normal morphology as surviving gametes, thus, they are the only ones with the right conditions for the resumption of meiosis. The maturation rate of these oocytes is between 44–55.6%, which is comparable to the fresh maturation rate of around 60% previously published in the domestic cat [28], showing that the procedure itself of inserting and pulling out the oocytes in closed tubes is not damaging the oocytes, this data was also supported by the maturation rate obtained in the control of toxicity test with COCs (59.1%). That seems to point out that the oocytes that survive vitrification are in similar conditions to go on with maturation processes as the fresh ones. However, as explained before, this trend was not continued in cleavage and advanced embryo rates. Thus, the average rate of 17.5% of cleavage stays far away from the 60% obtained with fresh oocytes [28]. In the future, it might be needed to discard whether the tubes themselves or the combination of metal tubes with vitrification solutions is the origin of some embryotoxicity.

Fluorescence dyes are widely used in reproduction biology. In oocytes not only the nucleus can be stained [29] but also cortical granules [30], the cytoskeleton of the ooplasm [31] and of the cumulus cells [32], and the lipid content [33] to analyse several biological processes. The nuclear stage in cat oocytes is useful to give us information on the meiotic competence when no morphological sign can be seen. In this case, around one-third of the oocytes that did not present a polar body were able to resume meiosis until different stages (either GVBD or MI). It remains unclear why this mechanism was blocked and did not progress, whether the cumulus cells are too damaged to send the right signals to the ooplasm or whether the ooplasm or the nucleus are simply incompetent to respond to them and proceed with the polar body extrusion. Previous studies showed that cat oocytes that undergo IVM have lower levels of maturation factors [34] than those that mature in vivo. This situation combined with possible damages caused by the freezing procedure could lead to a higher impairment of the oocytes. On the other hand, if taking morphology data into account, it is possible to observe that 17 oocytes from the aluminium group had a normal morphology after warming but the polar body was not present. This is the same number of oocytes that presented GVBD or MI in the fluorescence assay, even though it cannot be assumed that the oocytes are the same. However, the numbers in silver and titanium are different, 14 oocytes from the silver group and 12 from titanium looked intact, but 16 and 15, respectively restarted the meiosis. Considering that oocytes classified with abnormal morphology, here referred to membrane damages or shrunken after warming, cannot go on with biological processes this would point out that some oocytes might have been in advanced stages of meiotic maturation even before vitrification. As recently suggested, it might be that some oocytes are further on maturation stages in the ovary and need a shorter time in vitro to complete the first meiotic division [35].

Aluminium tubes proved to have a toxic effect on the cat gametes, as observed in the sperm toxicity test where motility decreased by twice in the treatment than in the control. This hypothesis was also supported by the oocyte toxicity test, where the maturation rate was significantly lower in the oocytes preincubated in the tubes with the vitrification solution, and there was no embryo cleavage at all. It indicates that the CPAs are washing out some toxic components from the tubes. In fact, the relation of ethylene glycol-based solutions and aluminium corrosion has been long discussed in other fields [36]. While the mechanism of degradation is being elucidated [37], we can suppose that the deterioration of the material is producing a harmful environment for the sensitive female gametes. In our toxicity test, only 13% of the oocytes could go on maturation after being in contact with the vitrification solution and the tubes for 2 min. This percentage is lower than the 22.9% obtained after freezing, probably due to the fact that during vitrification the contact time between CPAs and aluminium was limited to 30 s, reducing also the corrosion time and minimizing toxic effects of the material.

Both silver and titanium presented no signs of a toxic effect, though the decrease in sperm motility on the toxicity test was only around 8% higher in the treatment than in the control. Nevertheless, silver is long known for its antibacterial properties [38], and recent publications showed several dysfunctions in different biological mechanisms such as an increase in oxidative stress [39] and also its potential embryotoxicity [40,41,42,43]. The mechanism of maturation could also be harmed since in this group the lowest maturation rate was obtained.

For decades, titanium has been considered an inert material [44]. Although there are no significant differences, a minor tendency to obtain higher results is observed here, not only in maturation but also in the cleavage rate. In Figure 2D, a clear cumulus expansion is observed after the IVM of the oocytes frozen in these tubes. According to the toxicity test, no harmful effect was found, as in previous publications, where no signs of toxicity were found in several analyses [42,45] and it has actually been recommended widely for use as an implant material [40].

## 5. Conclusions

In conclusion, we consider that it is worth studying the combination of self-pressurized rapid freezing of cat oocytes with our vitrification protocol in a deeper extent. These results demonstrate that surviving oocytes have similar rates of maturation than fresh oocytes. Even though toxicity factors need to be considered, no significant difference was found between the use of different tube materials. Although embryo development was compromised when using this technique, we are convinced that this can be improved by further modifying the handling procedure. One reason for the developmental decline might be an insufficient sealing of the rough titanium tubes, which could be overcome by the use of better micro tools or the application of longer tubes. To reduce the toxicity observed with aluminium, tubes could be siliconized or covered with some protein. The aspect of reducing the CPAs concentration when using self-pressurized vitrification was not explored sufficiently, and demands more detailed studies. Nevertheless, these results are beneficial for the use of this methodology not only for gamete rescue purposes but also to cryopreserve other cell types under field conditions.

## Figures and Tables

**Table 1:** Morphological classification after warming and recovery of the oocytes frozen in different metal tubes with the self-pressure method.

Tube Material	n Total Oocytes	n Normal Oocytes (%)	n Oocytes with Cryodamages	
		(n Mature Oocytes (%)) *	Membrane Damage (%)	Shape Not Recovered (%)
Aluminium	61	31 (50.8) (14 (22.9))	18 (29.5)	12 (19.7)
Silver	63	25 (39.7) (11 (17.5))	18 (28.6)	20 (31.7)
Titanium	65	27 (41.5) (15 (23.1))	23 (35.4)	15 (23.1)
**Average of all tubes**	189	83 (43.9) (40 (21.2))	59 (31.2)	47 (24.9)
Vitrification control **	143	- (35 (24.5))	-	-

No significant differences were found between the groups. * Maturation rate is calculated from the total number of oocytes per group. ** Vitrification control corresponds to the previous experiments with the standard vitrification procedure performed in cryotops. Bold: the average outcome of all of tube material.

**Table 2:** Results of fluorescence analysis of immature oocytes after warming and IVM. Oocytes are classified as those that remained in the GV stage or those that went on with the resumption of meiosis (GVBD or MI).

Tube Material	n Total Immature Oocytes	GV (%)	Progress on Meiosis		n Not Assessable (%)
			GVBD (%)	MI (%)	
Aluminium	47	26 (55.3)	11 (23.4)	6 (12.8)	4 (8.5)
Silver	52	35 (67.3)	10 (19.2)	6 (11.5)	1 (1.9)
Titanium	50	32 (64)	11 (22)	4 (8)	3 (6)
**Average of all tubes**	149	93 (62.4)	32 (21.5)	16 (10.7)	8 (5.4)

No significant differences were found between the groups. Bold: the average outcome of all of tube material.

**Table 3:** Developmental competence of the thawed oocytes presenting a normal morphology, after IVM and ICSI.

Tube Material	n Total Normal Oocytes after Thawing	n Mature Oocytes (%)	n Cleavage (%)	n Morulae (%)
Aluminium	31	14 (45.2)	2 (14.3)	0 (0)
Silver	25	11 (44)	2 (18.2)	0 (0)
Titanium	27	15 (55.6)	3 (20)	0 (0)
**Average of all tubes**	83	40 (48.2)	7 (17.5)	0 (0)

No significant differences were found between the groups. Bold: the average outcome of all of tube material.

## Data Availability

The data presented in this study are available on request from the corresponding author.
